# Kinase Inhibitor Library Screening Identifies the Cancer Therapeutic Sorafenib and Structurally Similar Compounds as Strong Inhibitors of the Fungal Pathogen *Histoplasma capsulatum*

**DOI:** 10.3390/antibiotics10101223

**Published:** 2021-10-08

**Authors:** Charlotte Berkes, Jimmy Franco, Maxx Lawson, Katelynn Brann, Jessica Mermelstein, Daniel Laverty, Allison Connors

**Affiliations:** 1 Department of Biology, Merrimack College, North Andover, MA 01845, USA; lawsonm@merrimack.edu (M.L.); doironk@merrimack.edu (K.B.); mermelsteinje@gmail.com (J.M.); lavertyd@merrimack.edu (D.L.); 2 Department of Chemistry and Biochemistry, Merrimack College, North Andover, MA 01845, USA; francoj@merrimack.edu (J.F.); connorsa@merrimack.edu (A.C.)

**Keywords:** *Histoplasma*, antifungal, Sorafenib, drug repurposing

## Abstract

*Histoplasma capsulatum* is a dimorphic fungal pathogen endemic to the midwestern and southern United States. It causes mycoses ranging from subclinical respiratory infections to severe systemic disease, and is of particular concern for immunocompromised patients in endemic areas. Clinical management of histoplasmosis relies on protracted regimens of antifungal drugs whose effectiveness can be limited by toxicity. In this study, we hypothesize that conserved biochemical signaling pathways in the eukaryotic domain can be leveraged to repurpose kinase inhibitors as antifungal compounds. We conducted a screen of two kinase inhibitor libraries to identify compounds inhibiting the growth of *Histoplasma capsulatum* in the pathogenic yeast form. Our approach identified seven compounds with an elongated hydrophobic polyaromatic structure, five of which share a molecular motif including a urea unit linking a halogenated benzene ring and a *para*-substituted polyaromatic group. The top hits include the cancer therapeutic Sorafenib, which inhibits growth of *Histoplasma* in vitro and in a macrophage infection model with low host cell cytotoxicity. Our results reveal the possibility of repurposing Sorafenib or derivatives thereof as therapy for histoplasmosis, and suggest that repurposing of libraries developed for human cellular targets may be a fruitful source of antifungal discovery.

## 1. Introduction

Treatment of systemic fungal infections presents a formidable challenge to the healthcare system. Recent advances in medicine have saved the lives of millions with diseases and conditions that were previously untreatable. However, these advances, including immunomodulatory therapies for cancer treatment, autoimmune disease, and organ transplantation have contributed to the increasing population of individuals with immunocompromised status who have a heightened susceptibility to fungal infection [1]. Invasive and/or systemic fungal infections carry a high cost in human and economic terms; in the United States alone in 2017, 4.5 billion USD was spent on the treatment of fungal infections requiring hospitalization [2]. Unfortunately, our arsenal of antifungal therapies has not expanded at a sufficient pace to meet the need.

The dimorphic fungal pathogens—*Histoplasma*, *Blastomyces*, *Coccidioides* and *Paracoccidioides*—are of particular clinical concern as these organisms infect and cause disease in both immunocompromised and healthy hosts. *Histoplasma capsulatum* is a dimorphic fungal pathogen and the etiologic agent of histoplasmosis, the most common fungal respiratory infection in the United States [3]. In endemic areas in the Midwestern U.S., the soil harbors a sporulating mold form of the organism that can be aerosolized and inhaled by mammalian hosts. Fungal spores germinate within the lungs and establish growth as budding yeasts, which are subsequently phagocytosed by alveolar macrophages, one of the primary host cells for *H. capsulatum* during infection. *H. capsulatum* yeasts replicate within the macrophage phagosome, ultimately leading to macrophage lysis [3]. Control of the infection in healthy hosts requires a robust CD4^+^ response; therefore, individuals with impaired cellular immunity are at risk of developing severe disseminated disease. While the clearance of *Histoplasma* in healthy individuals is poorly understood, there is evidence that in some individuals the infection can enter a latent state with the potential to re-emerge following a period of weakened immunity [4,5,6,7].

Currently, the primary antifungals available for the treatment of serious and life-threatening fungal infections fall into just three mechanistic classes: the polyenes, azoles, and echinocandins. The polyene amphotericin B interacts with membrane ergosterol, thereby disrupting membrane integrity and acting in a fungicidal manner. Azole drugs function by interfering with ergosterol synthesis [8,9]. Treatment of acute and chronic pulmonary histoplasmosis as well as progressive disseminated disease relies on amphotericin B, sometimes followed by itraconazole [10]. The effectiveness of the deoxycholate formulation of amphotericin B is limited by renal and infusion-related toxicity, although toxicity has been partially mitigated by the development of liposomal formulations of amphotericin B [11,12]. The echinocandins inhibit synthesis of cell wall beta glucan and have been adopted for the treatment of disseminated candidiasis [13]. Unfortunately, *Histoplasma* and other dimorphic fungi harbor endogenous resistance to the echinocandins, limiting their effectiveness in the clinic [14,15].

A novel mechanistic class of antifungals has not been introduced to the clinic since the advent of the echinocandins two decades ago [8,16]. An expansion of the antifungal arsenal from which clinicians may draw from to treat patients with invasive fungal infections is long overdue. The barriers interfering with development of antifungal agents to treat invasive fungal infections are both scientific and economic/regulatory. Due in large part to the eukaryotic nature of fungi, the universe of structurally unique targets to be exploited for antifungal therapy is much smaller than for that of prokaryotic pathogens. Intracellular fungal pathogens such as *Cryptococcus* and *Histoplasma* present additional challenges in antifungal delivery due to the protected nature of their respective host cell niches. Antifungal compounds that are effective in vitro are unfortunately more likely than not to fail to clear regulatory hurdles. Drug repurposing has been an effective approach for a number of infectious disease targets [17]; the significant challenges of histoplasmosis treatment prompts consideration of a repurposing approach for *Histoplasma*.

In eukaryotic systems, protein phosphorylation switches play a central role in signaling pathways regulating nearly all cellular processes including proliferation, differentiation, and response to stress. Kinase inhibition has been a highly effective strategy for the development of anti-cancer therapies, and development of kinase inhibitor libraries optimized for drug-like qualities has supported and accelerated research in this area in recent years [18,19,20]. In the model yeast *Saccharomyces cerevisiae*, MAP kinase cascades have been shown to regulate response to osmotic stress, mating, cell wall integrity and ascospore formation [21,22,23,24,25]. Evidence from knockout studies in the model fungal pathogens *Candida albicans* [26,27,28] and *Cryptococcus neoformans* [29,30,31] have highlighted the roles of kinases in virulence. The conservation of protein kinase signaling throughout the eukaryotic domain suggests that these pathways may be pharmacologically modulated as a strategy to combat fungal and protozoan infections [32]. These strategies may employ kinase inhibitors to directly interfere with cellular machinery required for fungal virulence and/or viability, or may be used to amplify their effectiveness by interfering with antifungal resistance mechanisms [33].

In the current study, we screened two kinase inhibitor libraries to identify compounds inhibiting the growth of *Histoplasma capsulatum* in the pathogenic yeast form. From these libraries, we identified a set of seven compounds containing an elongated hydrophobic polyaromatic structural motif that inhibit growth of *Histoplasma capsulatum* in the low micromolar/nanomolar range. Five of the seven compounds contain highly similar molecular structures containing the chemical motif of a urea unit linking a halogenated benzene ring and a *para*-substituted polyaromatic group. One of these compounds, Sorafenib, is an FDA-approved compound currently used for treatment of renal small cell carcinomas. Sorafenib inhibits growth of *Histoplasma* both in vitro and in a macrophage infection model with low host cell cytotoxicity. Our work highlights the potential to repurpose Sorafenib as therapy for histoplasmosis, and identifies a molecular motif for further development.

## 2. Results

### 2.1. Kinase Inhibitor Library Screening Identifies Anti-Histoplasma Compounds

**Figure 1:**
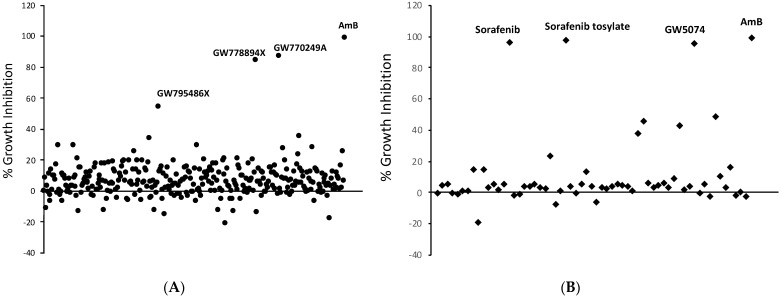
Graphical representation of primary screen results of the Published Kinase Inhibitor Set (**A**) and the SelleckChem MAPK Inhibitor Library (**B**) against *Histoplasma capsulatum*. Each individual compound was tested, in duplicate, at a final concentration of 5 μM. Log phase *Hc* WU15 yeasts were seeded in 96 well plates at a density of 1 × 10^6^ yeasts/mL in HMM/uracil. Compounds were diluted in DMSO and added to each well at a final DMSO concentration of 0.5%. Plates were incubated at 37 °C and 5% CO_2_ with twice-daily shaking to improve aeration. OD_595_ readings were taken on day 0 and day 4. The results for each compound are shown as average percent growth inhibition over the four day time period relative to the DMSO control. All compounds showing greater than 50% growth inhibition at day 4 are identified.

Two kinase inhibitor libraries were screened in an effort to identify novel molecules inhibiting *Histoplasma* growth. The GlaxoSmithKline Published Kinase Inhibitor Set (GSK PKIS) is a collection of 367 small molecule ATP-competitive kinase inhibitors optimized for drug-like physicochemical properties and developed for diverse kinase targets [34,35]. *Histoplasma capsulatum* WU15 (*Hc* WU15), a uracil auxotrophic strain derived from the clinical G217B isolate, was grown in microtiter plates in combination with GSK PKIS library compounds at a final concentration of 5 μM and growth was monitored over the course of four days by optical density determination as previously described [36,37]. Three compounds, GW778894X, GW770249A, and GW795486X, inhibited growth of *Hc* WU15 by greater than 50% relative to amphotericin B, with GW778894X and GW770249A displaying the most severe growth inhibition (Figure 1A). Given the evidence supporting a central role of fungal MAP kinase homologs in stress response and virulence, we also screened a commercially available library of 61 cell permeable inhibitors targeting MEK, Raf, p38, JNK, ERK and other MAP kinases. The MAP kinase library yielded three compounds meeting criteria for 50% growth inhibition over a four day time course as monitored by optical density: GW5074, Sorafenib, and the tosylate salt formulation of Sorafenib (Figure 1B).

**Figure 2:**
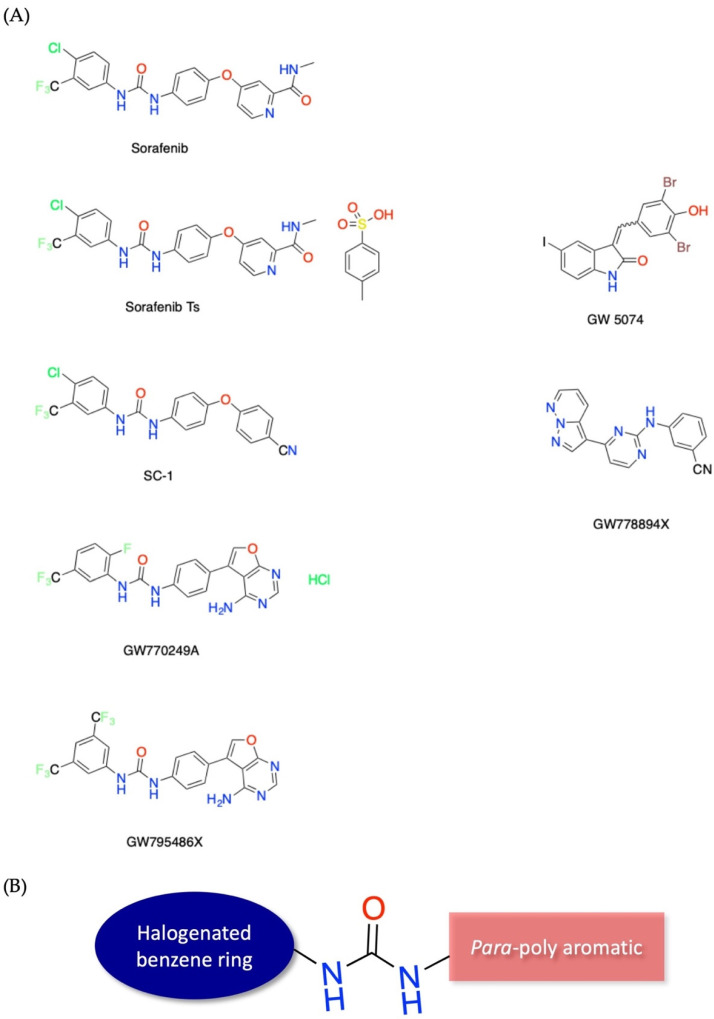
Chemical structures of compounds strongly inhibiting *H. capsulatum* growth at MIC < 2 μM. Sorafenib, Sorafenib tosylate, and GW5074 were identified in the SelleckChem library screen. SC−1 was included due to its structural similarity to Sorafenib. GW778894X, GW770249A, and GW795486X were identified in the PKIS library. (**A**) All seven of the compounds have an elongated hydrophobic polyaromatic structural motif. Five of the seven compounds contain highly similar molecular structures containing the chemical motif of a urea unit linking a halogenated benzene ring and a *para* substituted poly aromatic group, represented schematically in (**B**).

The chemical structures of top kinase inhibitor library hits are shown in Figure 2, highlighting the commonality of an elongated hydrophobic polyaromatic structural motif. Five of the six molecules identified in our screening efforts adhere to a structural motif containing a urea unit linking a halogenated benzene ring and a *para*-substituted polyaromatic group. The halogenated benzene portion of the molecule consists of either a F, Cl, or trifluoromethyl group, which are nonclassical bioisoteres of each other [38]. Additionally, most compounds exhibited favorable calculated logP values and satisfied Lipinski’s rule of five for drug-likeness [39,40].

### 2.2. Characterization of Sorafenib and SC−1 Antifungal Properties

**Figure 3:**
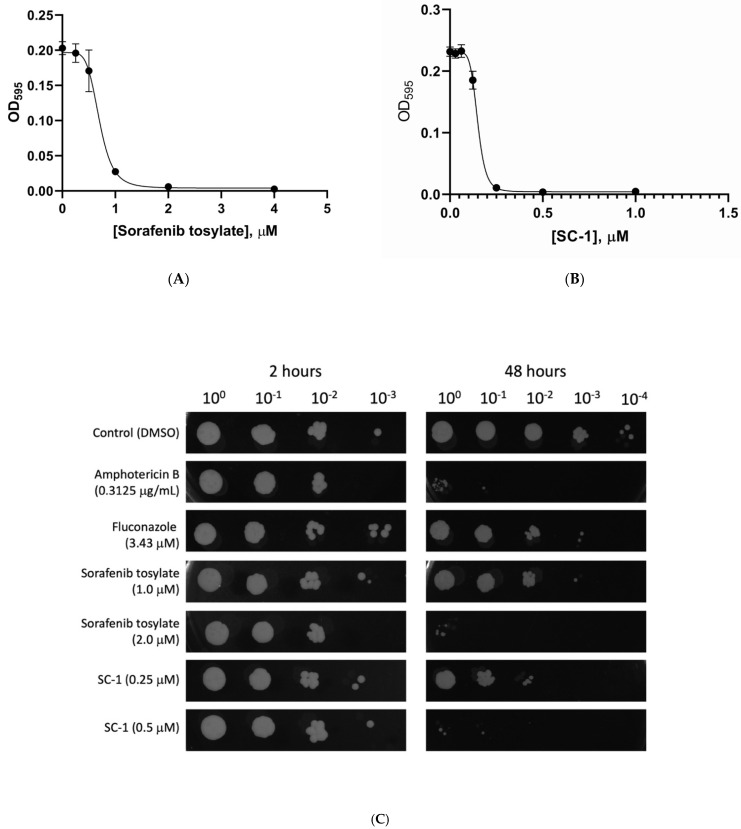
Sorafenib and SC−1 are fungistatic to *H. capsulatum* yeasts. *Hc* WU15 yeasts were seeded in 96 well plates at a density of 4 × 10^6^ yeasts/mL in HMM. Sorafenib tosylate (**a**) or SC−1 (**b**) were diluted in DMSO and added to each well at a final DMSO concentration of 0.5% with a two-fold dilution series of each compound. Growth of yeasts was monitored by measuring absorbance at 595 nm. IC_50_ values were calculated using nonlinear regression of the data at the 4 day time point. (**c**) Fungistasis was demonstrated by plating serial dilutions of yeasts onto solid media at 2 and 48 h following addition of compounds to measure the number of viable CFU. The images shown are representative of four independent experiments.

Sorafenib is an FDA approved multi-kinase inhibitor used for the treatment of renal cell carcinoma, hepatocellular carcinoma, and metastatic differentiated thyroid cancer. Sorafenib and its close structural derivative, SC−1 (devoid of Raf inhibitory activity), have both been shown to have anti-tumor and anti-fibrotic effects in a variety of cancer cell lines and in vivo models [41,42,43]. We hypothesized that SC−1 would also have a strong inhibitory effect on the growth of *H. capsulatum* based on the fact that it shares the same diarylurea structural motif as many of our kinase library screen hits. MIC and IC_50_ values for Sorafenib tosylate and SC−1 were determined by exposing *Hc* WU15 to two-fold serial dilutions of Sorafenib tosylate or SC−1 using protocols established previously for *H. capsulaum* G217B yeast form cultures [36,37]. Multiple dose response experiments determined the IC_50_ of Sorafenib tosylate and SC−1 are 0.69 μM and 0.15 μM, respectively (Figure 3A,B). The MIC values for Sorafenib tosylate and SC−1 are 1.0 μM and 0.25 μM, respectively (Figure 3 and Table 1).

To determine the spectrum of antifungal activity, we tested all compounds identified in the library screening experiments, as well as SC−1, against additional clinically relevant and model fungi (Table 1). None of the compounds interfered with growth of *Saccharomyces cerevisiae* or *Candida albicans* up to the highest concentration tested (50 μM). However, Sorafenib and SC−1 showed weak activity against *Cryptococcus neoformans* (Table 1). *H. capsulatum* G186AR is a “rough” isolate that is phylogenetically distinct from G217B. We tested the compounds against a uracil auxotrophic strain of *H. capsulatum* G186AR (*Hc* WU8), and found similar MIC values as for WU15.

To determine whether Sorafenib and SC−1 function in a fungistatic or fungicidal manner, we cultured *Hc* WU15 in media containing these compounds for 2 h and 48 h, then plated cultures on drug-free solid media. Additional wells contained amphotericin B or fluconazole, which inhibit *H. capsulatum* via fungicidal and fungistatic mechanisms, respectively. We used drug concentrations at the previously published MIC for amphotericin B (0.3125 μg/mL) and fluconazole (3.43 μM); we tested Sorafenib tosylate and SC−1 at their respective MICs (1.0 μM and 0.25 μM) and at double the MIC values 2.0 μM and 0.5 μM, respectively). Interestingly, both Sorafenib and SC−1 act in a fungistatic manner at the MIC concentration, whereas at 2× MIC the antifungal activity is fungicidal (Figure 3C).

To determine whether Sorafenib and/or SC−1 act synergistically with traditional antifungals, we performed checkerboard assays in which Sorafenib or SC−1 was added to *Hc* WU15 cultures in combination with fluconazole at a range of concentrations. In these experiments, we failed to observe synergy between Sorafenib or SC−1 and fluconazole (Supplementary File).

### 2.3. Effectiveness of Sorafenib and SC−1 against Intracellular H. capsulatum

**Figure 4:**
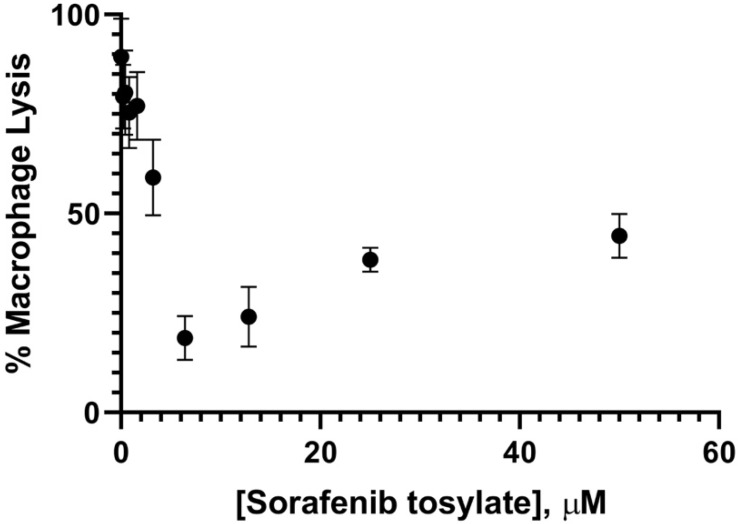
Sorafenib inhibits growth of intracellular *H. capsulatum* and macrophage lysis. Murine BMDM were seeded in 24 well tissue culture treated wells and infected with *Hc* WU15 at an MOI of 5. After two hours, extracellular yeasts were removed by washing and media containing Sorafenib-tosylate or DMSO only was added, with each condition in triplicate. Following 48 h incubation at 37 °C and 5% CO_2_, macrophage lysis was monitored by CytoTox LDH release assay. % lysis was calculated relative to a detergent-treated uninfected control. Data shown are representative of three independent experiments.

During an infection, *H. capsulatum* cells reside within the phagosomal compartment of alveolar macrophages where they persist and evade immune clearance. Therefore, anti-*Histoplasma* compounds must be able to target intraphagosomal yeasts in order to be effective. *H. capsulatum* replication within the macrophage phagosome culminates in macrophage lysis, and the ability of yeasts to stimulate lysis is required for virulence in vivo [44]. To further explore the potential to repurpose Sorafenib for antifungal therapy, we tested the ability of Sorafenib to inhibit lysis of host macrophages. Murine bone marrow-derived macrophages (BMDM) were infected with *H. capsulatum* at a multiplicity of infection of 5. After co-incubation of yeasts with BMDM for 2 h, extracellular yeasts were removed by washing with media and cultures were treated with DMSO or Sorafenib tosylate at a range of concentrations. The extent of macrophage lysis was determined by monitoring release of lactate dehydrogenase (LDH). At 48 h following infection, visual inspection of BMDM cultures treated with DMSO showed robust yeast growth culminating in BMDM lysis, as indicated by release of LDH into the culture media. Samples treated with Sorafenib tosylate showed a dose-dependent decrease in BMDM lysis up to 6.25 μM (Figure 4). Consistent with previously demonstrated cytotoxicity in mammalian cells at concentrations greater than 5–10 μM, wells treated with higher Sorafenib tosylate concentrations showed increased lysis (Figure 4). Taken together, these results are consistent with effective delivery of Sorafenib to the macrophage phagosome.

## 3. Discussion

The specific aim of this study was to identify kinase inhibitors with anti-*Histoplasma* activity, an approach that has previously been successful for both fungal and protozoan pathogens [45,46,47,48]. Our approach is based on a repurposing strategy in which conservation of kinase mediated signaling pathways in all eukaryotes may be leveraged to identify compounds within libraries optimized for drug-like qualities. Here, we present an adaptation of antifungal screening methods developed for the pathogenic yeast form of *Histoplasma capsulatum* [36,37] that utilizes an avirulent uracil auxotrophic strain cultured in media supplemented with uracil. We use this method to screen two kinase inhibitor libraries for compounds inhibiting *Histoplasma* growth. have an elongated polyaromatic motif and calculated log P values of 5.6 or less, which is generally considered drug-like and beneficial for absorption and permeability [39,40]. Similar to the recently identified aminothiazole derivatives demonstrating activity against *Histoplasma capsulatum*, the compounds identified here contain a central amide with cyclic hydrophobic groups on either side of the amide [36]. They also have predominantly elongated motifs, similarly to previously identified aminothiazole derivatives, which displayed greater activity when substituted at the 2,5 position of the aminothiazole ring, when compared to the 3 and 4 position [49]. Significantly, our approach identified the cancer therapeutic Sorafenib as a robust inhibitor of *Histoplasma capsulatum* growth.

Sorafenib and several of the compounds identified in our screen are diarylureas, which has served as an important molecular scaffold in anticancer drug development [50,51]. A growing body of work shows a strong potential for the use of the diarylurea scaffold in the development of antiviral, antiparasite, antibacterial and antifungal compounds [52,53,54]. Sorafenib in particular is reported to possess activity against drug-resistant *Staphylococcus aureus* and to interfere with replication of New World alphaviruses through a mechanism that is independent of host cell kinase inhibition [55]. Others have shown that other diarylureas possess antifungal activity [56], but ours is the first report that Sorafenib and SC-1 possess antifungal activity.

More generally, our work adds to a growing body of literature that has demonstrated the dual nature of anti-cancer or immunomodulatory compounds as antifungals [47,52]. The topoisomerase I-targeting compounds camptothecin and nitidine are active against *Cryptococcus* and *Saccharomyces*, and an exploration of structure-activity relationships for these drugs has identified chemical moieties associated with higher fungal selectivity [57]. The immunosuppressant rapamycin is also a potent antifungal agent whose activity is mediated via association with peptidylprolyl isomerase FKBP12, a target broadly conserved in both yeast and mammalian cells that forms a toxic complex with the TOR1 and TOR2 proteins in yeast or mTOR1 and mTOR2 proteins in mammals [58]. Wortmannin, a powerful immunosuppressant that acts via inhibition of PI−3 kinase in mammalian cells, exhibits antifungal activity that may be mediated by PI−4 and additional targets in yeast cells [59].

Sorafenib (BAY−43−9006 Nexavar, Bayer Pharmaceuticals Corp. and Onyx Pharmaceuticals Inc.) is a chemotherapeutic indicated for the treatment of hepatocellular carcinoma (HCC), renal cell carcinoma, and metastatic differentiated thyroid cancer. It is an orally active multi-kinase inhibitor that acts as a cytotoxic chemotherapeutic, inhibiting tumor cell proliferation and inducing apoptotic cell death via inhibition of VEGF and PDGF receptors as well as the Raf/MEK/ERK pathway [60]. SC−1 is a Sorafenib derivative lacking the functional amide group that confers Raf kinase inhibition activity upon Sorafenib. SC−1 is, however, a potent apoptosis inducer, functioning via a mechanism that includes inhibition of pSTAT3. The cytotoxicities of Sorafenib and its derivative, SC−1, on HepG2 and other human cell lines have been documented previously [41,42,43,61,62].

In yeasts, the high osmolarity-glycerol (HOG) pathway is a MAP kinase-mediated cascade controlling critical aspects of stress response including osmoregulation and cell wall integrity (reviewed in [63]). This pathway has been demonstrated to be a feasible target for antifungal development in a classic study of the antifungal compound ambruticin, which demonstrates its action via inhibition of the high osmolarity-glycerol pathway in the yeast *Hansenula anomala* [64]. Our results do not directly show that Sorafenib and/or SC−1 mediate growth inhibition in *Histoplasma* by mechanisms parallel to those demonstrated in mammalian cells. However, it is interesting to note that one of the human targets of Sorafenib, p38, displays 52% identity and 69% similarity at the amino acid level with its *H. capsulatum* homolog, Hog1. Further studies are needed to explore the mechanism by which Sorafenib and SC−1 mediate fungistasis in *H. capsulatum*.

## 4. Conclusions

The results of this study show that Sorafenib, SC−1, and structurally similar diarylureas inhibit the growth of *H. capsulatum* in the low micromolar range. Our study expands upon a previous literature demonstrating the potential use of antineoplastic drugs as antifungal agents, and is significant in that it specifically identifies the potential to repurpose Sorafenib and/or its derivatives as antifungal therapies. Furthermore, our findings support the concept of kinase inhibitor repurposing for fungal targets. Future work will establish a structure-activity relationship and optimize the effectiveness of Sorafenib derivatives for inhibition of *Histoplasma*.

## 5. Materials and Methods

### 5.1. Fungal Strains and Culture

*H. caspsulatum* WU15 (G217B *ura5*∆) and WU8 (G186AR *ura5*∆) have been described previously [65]. In uracil-replete media, G217B and WU15 display comparable growth rates and kinetics of macrophage lysis [44,66]. *H. caspsulatum* WU15 and WU8 cultures were maintained in yeast phase by culturing in liquid *Histoplasma* macrophage media (HMM) supplemented with 200 μg/mL uracil (Sigma-Aldrich, St. Louis, MO, USA) at 37 °C and 5% CO_2_ with constant agitation. *Candida albicans* SC5314 (ATCC MYA−2876), *Saccharomyces cerevisiae* BY4741 (ATCC 201388), and *Cryptococcus neoformans* H99 (ATCC 208821) were maintained in liquid or solid YPD media at 30 °C.

### 5.2. Chemical Library Screening

The GlaxoSmithKline Published Kinase Inhibitor Set (PKIS) was supplied by the University of North Carolina Structural Genomics Consortium under an open access Material Transfer and Trust Agreement. The PKIS library and the MAP kinase inhibitor library L3400 (SelleckChem, Houston, TX, USA) were provided as 1 mM stocks in DMSO. Immediately prior to experiments with *H. capsulatum*, 2× working stocks of compounds were prepared in HMM media. *H. capsulatum* cultures were prepared as previously described [36,37]. Briefly, overnight cultures in log phase were counted using a hemacytometer. Yeasts were seeded at 1.0 × 10^6^ yeasts/mL in a final volume of 50 μL, to which 50 μL of the kinase inhibitor working solutions were added such that the final drug concentrations were 5 μM and the final DMSO concentration was 0.5%. The negative control consisted of yeasts in 0.5% DMSO only and the positive control consisted of 0.3125 μg/mL amphotericin B (Sigma-Aldrich, St. Louis, MO, USA). Each library compound and control was tested in duplicate. Cultures were maintained in HMM-Ura at 37 °C and 5% CO_2_ with agitation twice daily. Growth was measured at 0 days and 4 days by measuring optical density at 595 nm (A_595_) using an EL800 plate reader (BioTek Instruments, Winooski, VT, USA). Percent inhibition was calculated relative to cells treated with DMSO vehicle using the following formula: [1 − (ΔOD_Test_/ΔOD_DMSO_)] × 100, where ΔOD_Test_ = OD_595_ at day 4 minus OD_595_ at day zero. For the DMSO control, ΔOD_Test_ = ΔOD_DMSO_, setting the % inhibition at 0%.

### 5.3. MIC Determination

The MIC of top hits identified in the library screens was determined for *H. capsulatum* WU15 yeast cells using the methods described previously [37]. *WU15* yeasts were prepared as for library screening and combined with serial 2-fold dilutions of each compound in 96 well microplates. Drug concentrations ranged from 0.03 μM to 4.0 μM. Cultures were maintained at 37 °C and 5% CO_2_ with agitation twice daily. Minimum concentrations that inhibited 100% growth relative to the day zero baseline were determined by visual inspection and confirmed by optical density measurement at 595 nm. Similar procedures were used for MIC determination using the WU8 strain, but MIC was determined using only visual inspection due to the clumpy nature of this strain relative to WU15. MICs for *Candida albicans* SC5314, *Saccharomyces cerevisiae*, and *Cryptococcus neoformans* H99 were determined as described previously [67]. IC_50_ values were calculated using non-linear regression with GraphPad Prism for macOS, Version 8.4.3, San Diego, California, USA.

### 5.4. LogP Determination

LogP values of inhibitory compounds were calculated using XLogP3, Version3.0. (PubChem release 2021.05.07): http://www.sioc-ccbg.ac.cn/skins/ccbgwebsite/software/xlogp3/ (accessed on 26 August 2019) [68].

### 5.5. Fungistatic/Fungicidal Testing

To monitor the mechanism by which Sorafenib and SC−1 inhibit *H. capsulatum* growth, cultures were set up using methods identical to MIC determination, using drug concentrations at the MIC or twice the MIC values. For comparison, cells were combined with amphotericin B or fluconazole. Cultures were incubated at 37 °C and 5% CO_2_. At two hours and 48 h following addition of compounds, samples were processed by performing serial dilutions in HMM/uracil liquid media. Three microliters of each dilution was spotted onto HMM agarose plates supplemented with 400 μg/mL uracil. Plates were allowed to incubate at 37 °C and 5% CO_2_ for six days, at which point colonies are visible. Four independent experiments were performed. Fungicidal mode of action was identified as a two-log reduction in colonies at the 48 h time point relative to the 2 h time point.

### 5.6. Macrophage Lysis Assay

Bone marrow-derived macrophages (BMDM) from 8 week-old female C57/Bl6 mice (a gift from Hooke Laboratories, Lawrence, MA, USA) were isolated from marrow differentiated in the presence of macrophage colony stimulating factor (CSF−1) as previously described [66]. Macrophage infections and lactate dehydrogenase (LDH) release assays were carried out using BMDM media [Dulbecco’s modified eagle medium (DMEM) without phenol red (Gibco-ThermoFisher, Waltham, MA, USA) supplemented with 20% fetal bovine serum (Cytiva HyClone, Marlborough, MA, USA) 2 mM glutamine, 1 mM Na-pyruvate, 100 U/mL penicillin, 100 μg/mL streptomycin, 400 μg/mL uracil and 20 ng/mL M-CSF (Gibco-ThermoFisher, Waltham, MA, USA). Phenol red was omitted from the media due to interference with the detection wavelength used for the LDH release assay. BMDM were seeded at a density of 2 × 10^5^ cells/well in 24-well tissue culture treated dishes (Corning- CoStar, Cambridge, MA, USA) and allowed to adhere for two hours. Separately, log phase *H. capsulatum* WU15 yeast cultures were pelleted, resuspended in DMEM without phenol red and counted by hemacytometer. Yeasts were added to macrophage wells at a multiplicity of infection (MOI) of 5. After a 2 h incubation period to allow for yeasts to be phagocytosed, the media was removed and macrophage monolayers were washed twice with DMEM without phenol red. Following macrophage washing, 750 µL BMDM media was added to each well. The infected macrophages were incubated at 37 °C with 5% CO_2._ At 24 h post-infection, 250 µL fresh media was added to each well. At 48 h following infection, LDH levels in BMDM supernatants were measured using the CytoTox Non-Radioactive cytotoxicity kit (Promega, Madison, WI, USA) as previously described [44,66]. The % lysis at each time point was calculated relative to LDH levels measured in uninfected BMDM artificially induced to lyse using 1% Triton X−100. Three independent experiments were performed.

## Figures and Tables

**Table 1:** Minimum inhibitory concentration (MIC) for top library screen hits against *Histoplasma capsulatum* WU15 and WU8, *Cryptococcus neoformans* H99, and *Candida albicans* SC5314.

Library	Compound	*Hc* WU15	*Hc* WU8	*C. neoformans*	*C. albicans*	Log P	Satisfies Lipinski’s Rule of 5?
	GW770249A	0.5	2.0	>50	>50	3.65	Yes
PKIS	GW7788994X	0.25	1.0	>50	>50	1.21	Yes
	GW795486X	2.0	4.0	>50	>50	4.41	Yes
	GW5074	2.0	4.0	>50	>50	5.31	Partially
SelleckChem	Sorafenib tosylate	1.0	2.0	25	>50	3.76	Yes
	Sorafenib	1.0	2.0	25	>50	3.8	Yes
-	SC-1	0.25	1.0	12.5	>50	5.56	Partially

## Data Availability

Data is contained within the article and supplementary material.

## Supplementary Materials

The following are available online at https://www.mdpi.com/article/10.3390/antibiotics10101223/s1, File: Sorafenib and SC−1 do not act synergistically with fluconazole or amphotericin B.
